# Toward Continuous Social Phenotyping: Analyzing Gaze Patterns in an Emotion Recognition Task for Children With Autism Through Wearable Smart Glasses

**DOI:** 10.2196/13810

**Published:** 2020-04-22

**Authors:** Anish Nag, Nick Haber, Catalin Voss, Serena Tamura, Jena Daniels, Jeffrey Ma, Bryan Chiang, Shasta Ramachandran, Jessey Schwartz, Terry Winograd, Carl Feinstein, Dennis P Wall

**Affiliations:** 1 University of California Berkeley, CA United States; 2 Graduate School of Education Stanford University Stanford, CA United States; 3 Department of Computer Science Stanford University Stanford, CA United States; 4 UC San Francisco San Francisco, CA United States; 5 Medable, Inc Palo Alto, CA United States; 6 Departments of Pediatrics, Biomedical Data Science, Psychiatry and Behavioral Sciences Stanford University Stanford, CA United States

**Keywords:** autism spectrum disorder, translational medicine, eye tracking, wearable technologies, artificial intelligence, machine learning, precision health, digital therapy

## Abstract

**Background:**

Several studies have shown that facial attention differs in children with autism. Measuring eye gaze and emotion recognition in children with autism is challenging, as standard clinical assessments must be delivered in clinical settings by a trained clinician. Wearable technologies may be able to bring eye gaze and emotion recognition into natural social interactions and settings.

**Objective:**

This study aimed to test: (1) the feasibility of tracking gaze using wearable smart glasses during a facial expression recognition task and (2) the ability of these gaze-tracking data, together with facial expression recognition responses, to distinguish children with autism from neurotypical controls (NCs).

**Methods:**

We compared the eye gaze and emotion recognition patterns of 16 children with autism spectrum disorder (ASD) and 17 children without ASD via wearable smart glasses fitted with a custom eye tracker. Children identified static facial expressions of images presented on a computer screen along with nonsocial distractors while wearing Google Glass and the eye tracker. Faces were presented in three trials, during one of which children received feedback in the form of the correct classification. We employed hybrid human-labeling and computer vision–enabled methods for pupil tracking and world–gaze translation calibration. We analyzed the impact of gaze and emotion recognition features in a prediction task aiming to distinguish children with ASD from NC participants.

**Results:**

Gaze and emotion recognition patterns enabled the training of a classifier that distinguished ASD and NC groups. However, it was unable to significantly outperform other classifiers that used only age and gender features, suggesting that further work is necessary to disentangle these effects.

**Conclusions:**

Although wearable smart glasses show promise in identifying subtle differences in gaze tracking and emotion recognition patterns in children with and without ASD, the present form factor and data do not allow for these differences to be reliably exploited by machine learning systems. Resolving these challenges will be an important step toward continuous tracking of the ASD phenotype.

## Introduction

### Background

Autism Spectrum Disorder (ASD) continues to be one of the most important public health challenges we face today, with 1 in 59 American children affected by it [1-3]. Children with autism are well known to differ from neurotypical controls (NCs) in their emotion recognition and facial processing patterns [4-9]. There are several leading theories about facial processing in ASD, and the underlying biological mechanisms are not fully understood [10-12]. However, children with autism exhibit many observable symptoms in facial attention, such as a lack of eye fixation, increased fixation on mouths [13], and requiring more time to extract emotions from faces [14]. Prior studies have found that individuals with autism have particular trouble recognizing certain emotions [15], such as happiness, neutrality [16], surprise [13,17-19], and fear [16,20]. At a more abstract level, they have been shown to struggle with making complex social judgements about trustworthiness, shame, and approachability [8]. These eye contact and facial affect recognition skills are important to improve social functioning [5], but the methods currently used to measure and track such skills are delivered in clinical settings or via trained administrators [21,22] outside of the social context where these skills are practiced.

Measuring emotion recognition and eye gaze in children with autism through mobile and wearable machine learning platforms has the potential to fill this gap for continuous phenotyping [23-28] during natural social interactions. Thus far, emotion recognition [8,16,20] and social attention [29,30] in autism have mostly been studied in isolation. Both have separately been proposed as indicators for diagnosis and quantification of autism. We hypothesized that they are deeply linked and studied them together as potential markers for phenotyping using wearable smart glasses and eye tracking.

### Objectives

In this study, we compared gaze and emotion recognition pattern data from 16 children with ASD to 17 NCs participating in an in-lab computer-based emotion recognition task to determine if gaze differences exist between ASD and NC children. Participating children were tasked with identifying emotions of standardized faces on a computer screen. During the task, they wore an early prototype of a Google Glass–based emotion recognition learning aid named *Superpower Glass* [9,23,31-34], fitted with a custom-built eye tracker that followed children’s gaze looking at emotional stimuli or distractors. This prototype is one of several attempts [35-37] to use Google Glass as a learning aid for children with ASD. A prior analysis focusing only on the emotion recognition data obtained from this study showed that ASD and NC participants differed only subtly in emotion recognition accuracies but that participants from the two groups showed noticeably different patterns in their emotion responses [31]. Children were eager to engage with the smart glasses, showing promise for the form factor. In analyzing gaze-tracking data from this study, we aimed to explore whether combining gaze and emotion recognition data may yield better distinguishing features for the two groups. We hypothesized that (1) the NC and ASD groups differ in gaze attention patterns and (2) this difference enables us to design an interpretable machine learning classifier distinguishing the two groups on our wearable platform.

## Methods

### Participants

Families were recruited from February 25, 2015, to January 26, 2016, at Stanford University via the Autism and Developmental Disabilities Research Registry, referrals to the Autism and Developmental Disabilities Clinic, the Developmental Behavioral Unit of Lucile Packard Children’s Hospital, and through academic presentations. ASD participants were included if they were between the ages of 6 to 17 years and if they provided an official autism diagnosis confirmed via medical record. We assessed parent reports of each child’s diagnosis via the Social Communication Questionnaire (SCQ) [38]. Participants are screened positively for ASD if they scored >16 on the SCQ. ASD participants were excluded if they had (1) evidence of a genetic, metabolic, or infectious etiology for their autism (in other words, had *syndromic autism*) based on medical record; (2) history of seizures or other neurologic disorders; (3) vision impairment; and/or (4) history of personality or bipolar disorder. NC participants were excluded if they had any of the following: (1) a score >14 on the SCQ, (2) a history of mood or personality disorder confirmed via parent report or medical record, (3) a sibling diagnosed with ASD or schizophrenia, (4) a history of seizures and/or other neurologic disorder, or (5) vision impairment.

### Procedure

Eligible participants (both parents and children) provided written informed consent under an approved institutional review board (IRB) protocol. Following consent, a trained research assistant delivered the Stanford Binet Intelligence Scales, Fifth Edition, Abbreviated Battery Intelligence Quotient (ABIQ) [39] to each child participant, and parents completed the Social Responsiveness Scale (SRS)-2 [40]. Demographic and evaluation results are demonstrated in Table 1.

**Figure 1:**
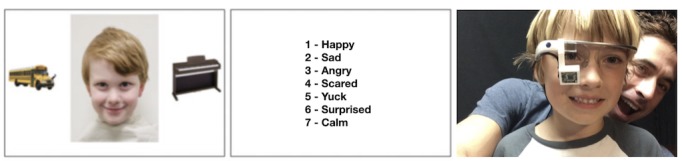
Study setup: (a) Study screen displaying facial affect stimuli and nonsocial distractors displayed for 6 seconds. (b) The screen displaying the list of emotions that the participant is asked to classify the face from. (c) A nonparticipant child wearing a Google Glass with a custom-built eye tracker fitted using a 3D-printed mount in a dry-run of the study protocol.

Participants wore Google Glass fitted with an eye tracker over the course of a 20-min computer task in which they were asked to identify the emotion (ie, happy, sad, angry, scared, disgust, surprised, and calm) portrayed by child actors on a screen. Before the task began, participants were familiarized with the list of facial emotions they would be asked to choose from. Participants were seated approximately 25 inches from the 24-inch screen (1920×1200 resolution) so that stimuli were presented at the eye level. Researchers conducted three successive trials using 125 images selected from the Child Affective Facial Expression (CAFE) dataset [41], balanced in each trial for race, gender, and emotion expression (T1 N=41, T2 N=42, and T3 N=41). The CAFE dataset is a set of diverse faces of children aged 2 to 8 years (mean 5.3 years, SD 1.5; range 2.7-8.7) depicting seven emotional facial expressions (ie, sad, happy, surprise, anger, disgust, fear, and neutral). The full set of 154 images includes 90 female and 64 male children that represent an even balance of African American, Asian, white, Latino, and South Asian racial groups [41]. Along with each facial affect image, each frame during all three trials displayed two nonsocial images of high autism interest (ie, Legos, train, and car) that have been previously validated [42] to its right and left to create an opportunity for distraction from the center emotion expression image (see Figure 1). Each facial stimulus covered approximately 49% of the width and 87% of the height of the screen. The two distractors were each displayed at approximately 17% screen width and 31% screen height.

Facial images and corresponding distractor images were displayed for 6 seconds before the participant was prompted to choose from a list of the seven possible emotions. The list of emotions was displayed until the participant verbally responded. The glasses were deactivated during the first and third trial. In the second trial, after 3 seconds of displaying the image, the glasses played an audio cue, speaking out the correct labeled emotion for the displayed image, emulating the emotion recognition functionality of the glasses. The first 4 participants received visual feedback in the form of a word shown on the heads-up display indicating the emotion, but this was found to be distracting, with reading ability strongly affecting behavior, and was replaced by auditory cues [9,31]. Between each trial, the eye tracker was recalibrated. Between every eight images within each trial, a dot displaying at its center a dancing Santa Claus appeared in the middle of the screen for 5 seconds to draw the child’s attention to the middle of the screen for validation of the eye tracking calibration. The sessions were all video recorded, and a researcher accompanied the participant through the task, recording the response option. Emotion classification responses were confirmed via the video session recording. During the second trial, participants received feedback from the glasses indicating the correct emotion classification.

**Table 1:** Cohort composition after excluding study failures. Medication/comorbidity surveys were not completed by 5 participants from the autism spectrum disorder cohort.

Demographic and phenotypic characteristics		Autism spectrum disorder (N=16)	Neurotypical controls (N=17)
**Gender, n (%)**			
	Males	13 (81)	9 (53)
	Females	3 (19)	8 (47)
Age (years), mean (SD; range)		12.13 (3.31; 6-17)	11.53 (2.48; 8-17)
Social Communication Questionnaire score, mean (SD; range)		18.86 (6.43; 7-31)	1.82 (1.07; 0-4)
Abbreviated Battery Intelligence Quotient standard score, mean (SD; range)		102.75 (19.54; 55-133)	108.94 (9.58; 91-129)
Social Responsiveness Scale Total score, mean (SD; range)		78.85 (11.13; 58->90)	44.41 (8.11; 36-64)
**Comorbid psychological conditions, n (%)**			
	Anxiety disorder/depression	1 (9)^a^	0 (0)
	Attention-deficit/hyperactivity disorder	1 (9)^a^	1 (5)
**Current medication, n (%)**			
	Methylphenidate	3 (27)^a^	0 (0)
	Arginine vasopressin	1 (9)^a^	0 (0)
	Guanfacine extended release	1 (9)^a^	0 (0)
	Sertraline	2 (18)^a^	0 (0)
	Carbamazepine	1 (9)^a^	0 (0)
	Aripiprazole	1 (9)^a^	0 (0)
	Dexmethylphenidate	1 (9)^a^	0 (0)
	Allergy medication (unspecified)	0 (0)^a^	1 (5)
	Other (unspecified)	0 (0)^a^	1 (5)
	No medication	4 (36)^a^	15 (88)

^a^N=11.

### Gaze-Tracking Apparatus

The Google Glass worn by the participant was fitted with a custom 3D-printed mount that slid onto the unit’s prism, holding a repurposed Microsoft LifeCam HD-6000 Webcam acting as an eye tracker (Figure 1). The webcam was modified by breaking and removing its infrared filter and replacing two indicator light emitting diodes with infrared emitters to produce a low-cost pupil recording device. The mount and modified webcam were adapted from the open source Pupil Project prototype [43]. A number of different mounts were 3D-printed such that at the beginning of each session, a mount could be chosen that provided the best view of the participant’s eye. The webcam was connected to the computer, displaying the facial stimuli, where outward-facing video from the Glass unit was recorded and synchronized with the inward-facing eye video. The total hardware cost of the eye tracker add-on was approximately US $35.

### Gaze-Tracking Data

In what follows, we have briefly outlined the procedure for obtaining gaze-tracking estimates. Details are given in Multimedia Appendix 1.

#### Pupil Tracking

We employed hybrid pupil identification methods, including maximally stable extremal regions [44], a gradient-based method [45] and an optical flow approach [46].

#### Calibration for World-Gaze Spatial Correspondence

Each participant performed four calibration processes, one at the beginning and one after each trial, yielding pupil coordinates (relative to the field of view of the eye-facing camera) and corresponding coordinates of the direction of gaze (referred to as *world-gaze coordinates* relative to the field of view of the front-facing camera). The true-value of the direction of each eye gaze event was assumed to be the direction of the stimulus provided on-screen, with no verification procedure. For each calibration, a separate polynomial regression was performed, yielding four candidate gaze prediction models. The gaze prediction models each provided, for each frame (image captured by camera), a predicted direction of eye gaze in world-gaze coordinates.

Each participant’s eye tracking session was then manually inspected and partitioned into time intervals for which of the four calibration models, or none, visually appeared to apply.

#### Gaze Clusters

Using the world-gaze coordinate estimate, we coded each frame as 1 of 4 categories: (F) on the facial stimulus, (L) on the left distractor stimulus, (R) on the right distractor stimulus, or (N) “nowhere in particular.” Calibration and gaze cluster labeling were semiautomated processes that were performed by independent labelers. Further details and interlabeler reliability data are provided in Multimedia Appendix 1.

#### Outlier Exclusion

In some instances, various failures (eg, camera slipping out of the mount made gaze tracking infeasible) led to unusable data. Trials with such data were discarded as outliers upon visual inspection. Detailed criteria are available in Multimedia Appendix 1.

### Hypothesis 1: Gaze Pattern Analysis

To test hypothesis 1, we calculated the following distraction ratio for each participant *p* and each facial stimulus *s* in each trial *t*:


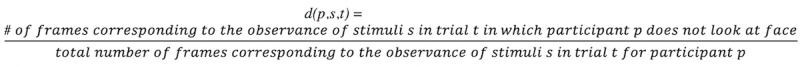


We plotted a histogram to visualize the differences between ASD and NC in gaze patterns throughout the task. We averaged the distraction ratios for multiple trials for each participant to arrive at a per-participant distraction ratio *d(p)*. We then performed 1-tailed *t* tests on the distraction ratio aggregated across groups (ASD vs NC) to test the primary hypothesis. We also performed exploratory *t* tests on the *d(p,s,t)*s across groups. However, this assumes what is likely an unreasonable noise model in which the *d(p,s,t)*s are independent within the same participant.

To make a more direct comparison between attention to face and attention to distractions, we further computed the given *p*, *s*, and *t* as mentioned earlier:


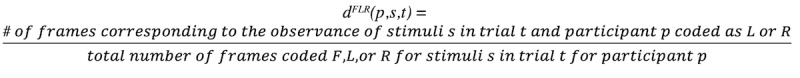


This excludes frames coded as N from the denominator. We then averaged *d^FLR^* over all stimuli in trial *t* corresponding to true-value emotion *e* to obtain *d_e_^FLR^(p,t)*.

### Hypothesis 2: Machine Learning Classification

To test hypothesis 2, we designed a machine learning classifier predicting binary ASD vs NC from the following features:

- Emotion confusion matrices (cm; 49-dimensional): 7×7 confusion matrices were computed for each participant across the entire trial, defined as the square matrix with rows and columns corresponding to the possible emotion responses, with entry in row *r* and column *c* to be the number of frames for which the true depicted emotion corresponds to *r*, but the participant inputted the emotion corresponding to *c* The confusion matrices were then normalized such that all rows summed to 1. Each element of the resulting normalized confusion matrix was extracted as an independent feature.
- Emotion confusion details (conf; 41- or 42-dimensional, depending on the trial): every face in the trial was assigned a binary value indicating whether the participant correctly identified the emotion, encapsulating the performance over time.
- Gaze patterns (gaze; 123- or 126-dimensional, depending on the trial): For the 6-second duration of eye tracking corresponding to a face in the trial, several features were extracted: the percentage of frames spent looking directly at the face as opposed to either of the distractors, percentage of changes of gaze fixations directed toward the face, and the number of frames elapsed before looking directly at the face. Each face contributed three of these additional features to the large pool of features.
- Participant metadata (pat; 2-dimensional): The participant’s age and gender were considered meta-features.

This yielded a total of 219 features per trial for consideration. As this number far exceeds the number of participants, regularization was important to prevent model overfitting. An elastic net model was chosen as the base classifier, as this model incorporates both lasso and ridge regularization. Our primary model, then, was an elastic net classifier trained and evaluated on all available trial data using all features, concatenated by trials. For various ablations, we trained elastic net classifiers and standard logistic regression classifiers on subsets of the features and the three trials individually. We evaluated accuracy across all classifiers using leave-one-participant-out cross-validation. We performed Monte Carlo shuffling tests to assess the statistical significance of classifier predictions. For logistic regression, we identified hyperparameters, including the type of regularization (*l*_1_ or *l*_2_), automatically using grid search on the training sets. Note that the logistic regression models are a special case of elastic net in which one of the regularizing terms is set to 0. In practice, optimizers for elastic net can perform unstably in these edge cases, and so we included the logistic regression model as a way of better optimizing over hyperparameters.

## Results

### Overview

Between February 25, 2015, and January 26, 2016, we enrolled 43 (ASD=23; NC=20) participants at Stanford University under a Stanford University IRB-approved protocol. We were unable to use data from 10 participants because of the following technical errors: 4 NC participants were excluded because they had received an early version of the Superpower Glass intervention, which included a visual display of the correct word describing the emotion displayed in the second trial but were unable to read those cues or complete the computer task. After realizing this failure, the prototype was adapted such that the remaining participants received only audio cues. Five ASD participants were excluded because of an image order randomization error as they received visual stimuli in a different order than all other participants. Finally, 1 ASD participant experienced a health issue during the study that was unrelated to the study procedures and was unable to complete study procedures.

The following analysis was conducted with 16 ASD participants (mean age 12.13, SD 3.3 years) of whom 81% (13/16) were male and 17 NC participants (mean age 11.53, SD 2.5 years) of whom 53% (9/17) were male. See Table 1 for additional participant demographics including SCQ, SRS, and ABIQ scores.

### Hypothesis 1: Gaze Pattern Analysis

Children with ASD showed a higher mean distraction ratio (mean 0.0433, SD 0.0911) than NCs (mean 0.0139, SD 0.0215), but this difference was not significant across the 33 participants in a 1-tailed *t* test (*P*=.12).

**Figure 2:**
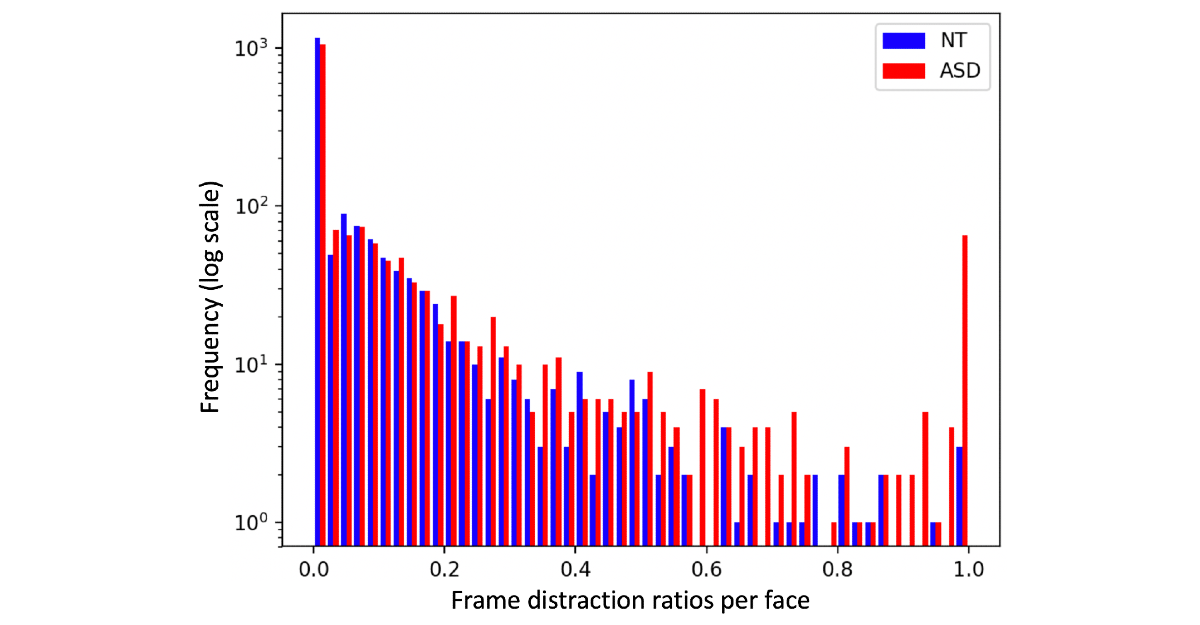
A histogram of the distraction ratio of autism spectrum disorder (ASD; red) and neurotypical control (NC; blue) participants on a logarithmic scale. On average, the ASD group looked at facial stimuli for less time than NCs. However, there is also considerable overlap between the groups that reduces the predictiveness of gaze features in the individual diagnosis prediction task.

When per-facial-stimuli distraction ratios *d(p,s,t)* were compared (1792 ASD samples and 1756 NC samples), a significant difference between ASD and NC datapoints was observed, making an independence assumption for the data within each of the two groups (uncorrected *P*<.01). A histogram of distraction ratios is shown in Figure 2 and reveals that distraction was higher and more inconsistent in participants with autism than in NCs.

**Figure 3:**
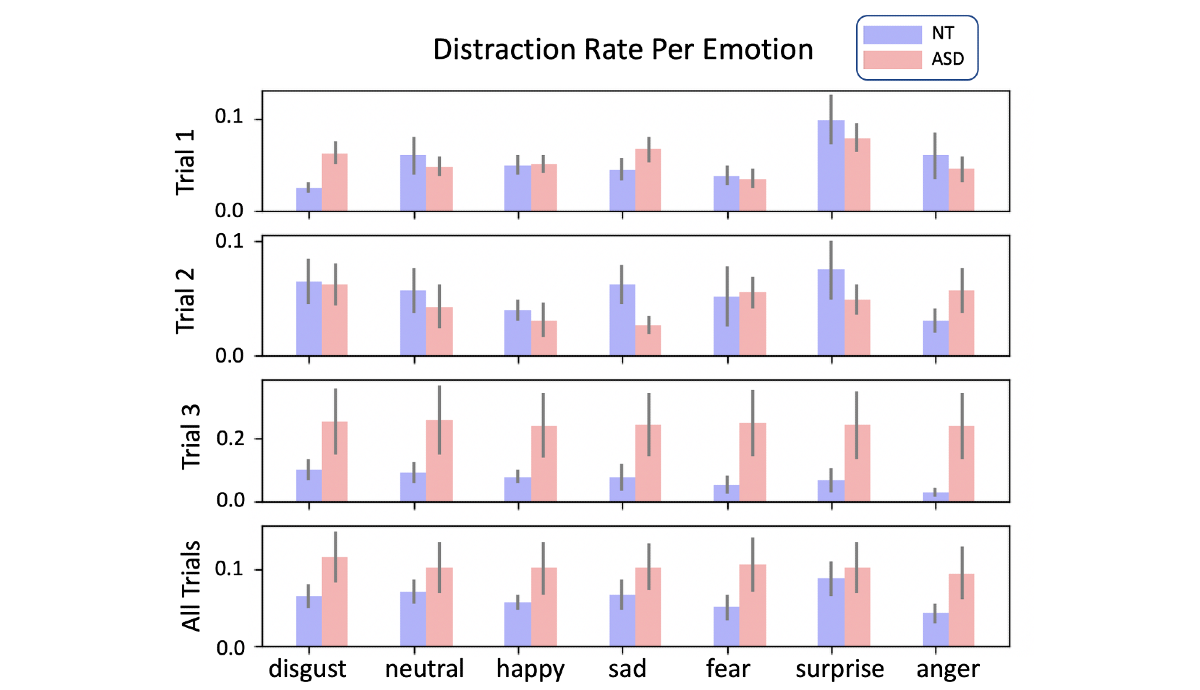
Histograms of (N-frame excluding) distraction ratio deFLR(p,t) of autism spectrum disorder (red) and neurotypical control (blue) participants, averaged over participants and broken down by emotion.

Similarly, we plotted N-frame-excluding distraction counts *d_e_^FLR^(p,t)*, averaged over all participants in the ASD and NC groups, keeping each emotion separate (Figure 3). Averaging over each emotion, we found no significant differences in the means between groups (*P*=.11 in trial 3, higher in others), owing largely to the high variance in the ASD group.

### Hypothesis 2: Machine Learning Classification

**Figure 4:**
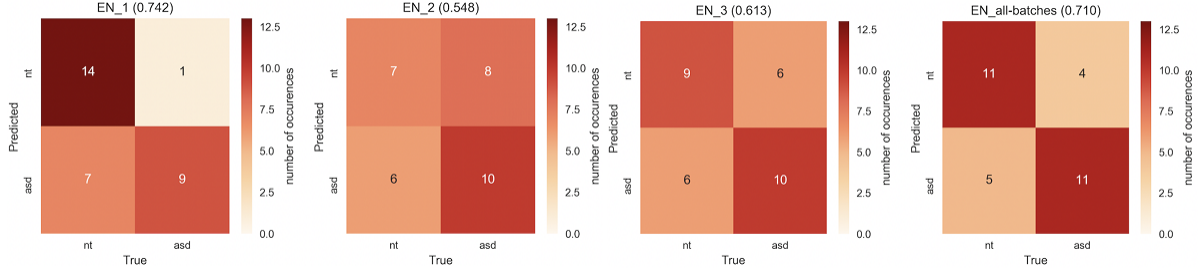
Cross-validation confusion matrices for the elastic net classifier trained on all features (pat, gaze, conf, and cm) for trials 1, 2, and 3, and features from all trials concatenated (accuracies in parenthesis). Autism spectrum disorder and neurotypical control participants are most distinguishable in trial 1, the first trial conducted which was before receiving any feedback or adjusting to the task. cm=Emotion confusion matrices; conf=Emotion confusion details; gaze=Gaze patterns; pat=Participant metadata.

**Figure 5:**
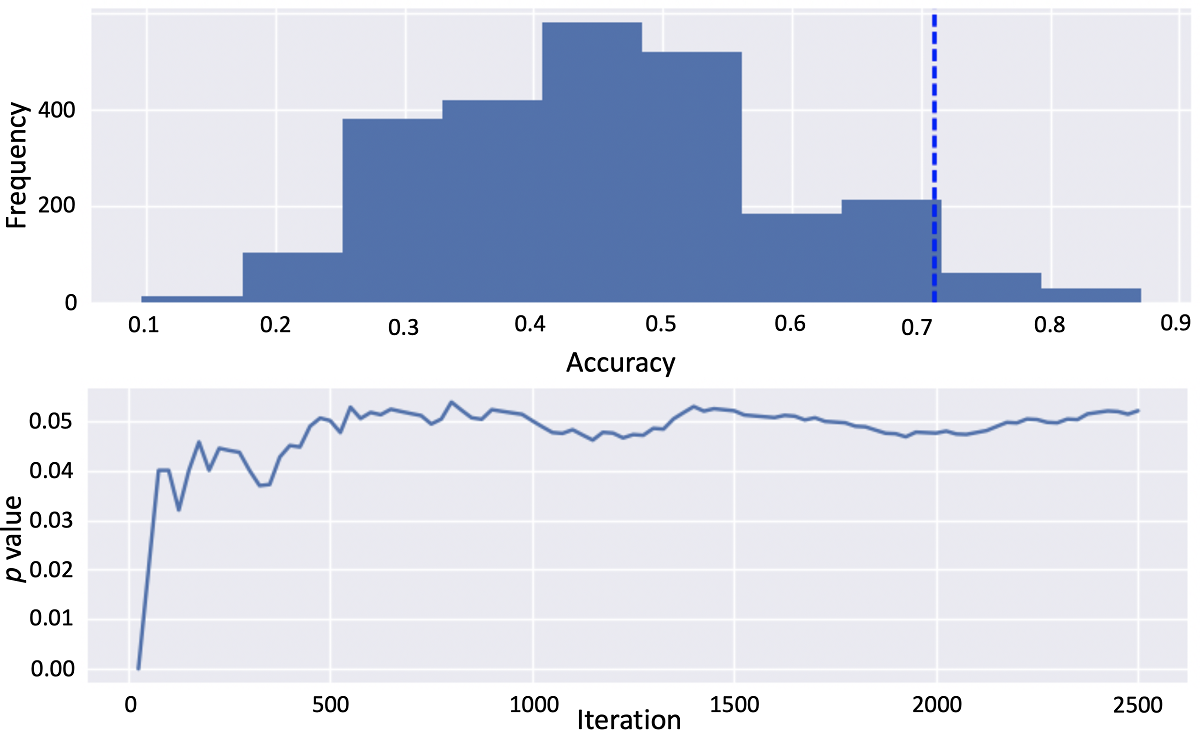
The shuffle test visualization for the elastic net classifier trained on all features (pat, gaze, conf, cm) concatenated for all trials yielding *P*=.05. cm=Emotion confusion matrices; conf=Emotion confusion details; gaze=Gaze patterns; pat=Participant metadata.

Cross-validation confusion matrices for an elastic net classifier trained on all features are presented in Figure 4. Across all trials, the model achieved a classification accuracy of 0.71 (*P*=.52 using Monte Carlo shuffling; see Figure 5).

Elastic net classification accuracies and uncorrected significance tests for all feature combinations are presented in Table 2. Classifiers trained on some gaze and expression recognition features were able to outperform those trained on combinations of pat, conf, and cm features in some cases, with significant *P* values on a shuffling test achieving significance before (but not after) a Bonferroni correction. Performance was best on all-trial features as well as data restricted to trial 1. However, given the relatively high performance on participant metadata (age and gender), we could not conclude that gaze and expression recognition features enhance classification. Elastic net models outperformed logistic regression ablation models in most tasks, including the primary pat-gaze-cm-conf model, suggesting a need for regularization in this dataset.

**Table 2:** Classification accuracies and significance tests for the elastic net classifier trained on different feature combinations and trials. All shuffle tests were performed for 2500 iterations and checked for convergence.

Data tested	All trials		Trial 1		Trial 2		Trial 3	
	Accuracy, %	*P* value	Accuracy, %	*P* value	Accuracy, %	*P* value	Accuracy, %	*P* value
pat^a^ (baseline)	71.0	.04	71.0	.04	71.0	.04	71.0	.04
pat-gaze-cm-conf (full)	71.0	.05	74.2	.03	54.8	.24	61.3	.17
cm^b^	67.7	.08	67.7	.09	6.5	.998	35.5	.81
conf^c^	64.5	.16	58.1	.23	48.4	.63	41.9	.72
cm-conf	64.5	.13	61.3	.17	48.4	.56	41.9	.68
gaze^d^	83.9	.002	83.9	.00	38.7	.80	61.3	.14
gaze-conf	83.9	.004	80.6	.01	51.6	.34	61.3	.17
gaze-cm	71.0	.05	64.5	.10	61.3	.14	58.1	.21
gaze-cm-conf	71.0	.05	74.2	.04	45.2	.63	51.6	.36
pat-gaze	77.4	.02	71.0	.04	67.7	.09	74.2	.03
pat-gaze-cm	71.0	.05	64.5	.11	67.7	.08	64.5	.11
pat-gaze-conf	74.2	.04	67.7	.11	35.5	.85	64.5	.14
pat-cm	58.1	.21	54.8	.25	67.7	.09	61.3	.16
pat-conf	64.5	.14	61.3	.19	64.5	.13	51.6	.35
pat-cm-conf	61.3	.17	48.4	.45	64.5	.13	32.3	.88

^a^pat: participant metadata.

^b^cm: emotion confusion matrices.

^c^conf: emotion confusion details.

^d^gaze: gaze patterns.

## Discussion

### Principal Findings

In this study, we compared gaze and emotion recognition pattern data of 16 children with ASD with 17 NCs. Participants completed an in-lab computer-based emotion recognition task, where they wore an early prototype of the Superpower Glass system [9,31,32], a Google Glass–based emotion recognition learning aid, as well as a custom-built eye tracker attached to the glass that followed children’s gaze looking at an emotional or distractor stimulus. In this limited data sample, we were unable to construct a machine learning classifier that reliably exploits these differences to predict ASD severity, to an accuracy significantly beyond that of the use of the age and gender data baseline. Although some models modestly outperformed a metadata-only baseline, prediction margins and significance did not hold up consistently across various ablations. Considering the large variance in gaze distraction in the ASD cohort, more data, perhaps more balanced for age and gender across cohorts, are likely required to develop a reliable model of greater use. A larger, more balanced corpus of data would also enable the use of more complex statistical models, such as artificial neural networks, which have the capacity to capture more complex structure within the data.

As an increasing amount of research work points to differences in facial attention in ASD [10-14], this study adds evidence that children with autism, when presented with facial stimuli relevant to a task involving social judgement, along with other, distracting stimuli, are more likely to attend to the distracting stimuli more. However, it also cautions that, just like with the literature on emotion recognition [16-20], this difference is subtle. Large and well-structured datasets are required to develop a diagnostic and phenotyping marker from this effect. Classical eye tracking studies have explored the subtlety of this effect in greater detail [30,47-52] and showed that it can vary depending on the emotional stimulus and setup. Some studies also observed that children with ASD pay more attention to the mouth than the eye region during an emotion recognition task [30,49-51]. The gaze data collected by our system was too noisy to test for these subtler effects. The prevailing belief in the literature was that facial attention differences are very clear in ASD, so we hypothesized that distraction ratios across an emotion recognition task alone were enough to distinguish ASD vs NC reliably. We now believe that integrating further subtlety through robust gaze-tracking hardware will be required to design a phenotyping marker from this effect.

### Limitations

Our study had a number of limitations including:

- *Limited age range*: The age range of participants was limited to 6 to 17 years in this study. Unfortunately, this age range is not representative of children who are in critical periods of development for cognition and speech, and therefore further feasibility testing on younger children is necessary.
- *Imbalanced gender ratio*: In this study, we observed a gender ratio of 13 males to 3 females in our ASD cohort. Though males are substantially more likely to be diagnosed with autism than females by an average ratio of 4 males:1 female, the reported imbalance still presents a gender bias between our sample population of children with ASD to our NC children. Furthermore, there are gender differences in both neurotypical and ASD children on emotion recognition and gaze tasks [15], which may have been exacerbated by our imbalanced cohorts.
- *Limited coverage of the autism spectrum*: Only 3 of our 16 ASD participants had an ABIQ lower than 80 (between 55 and 79), suggesting that 13 of our 16 recruited ASD participants can be classified as children with high-functioning ASD. This limits our findings, as their performance may not be reflective of children from across the autism spectrum.
- *Chinrest added after study started*: Due to too much head movement by the first 4 participants, we added a chinrest to provide additional stability for the remaining subjects. This was an issue especially because more ASD subjects took part in the study later on. This confound may be exploited by the classifiers.
- *Nonstandard eye tracking:* The eye tracking system used in this study was custom-built and has not been evaluated on a standard dataset or compared with standard eye trackers. The mount and camera shifted during the study, requiring recalibration. A series of manual checks were employed to correct for these issues and ultimately manually annotate much of the pupil and world-gaze coordinates, as described in Multimedia Appendix 1. The mount occluded roughly 15% of the field of view in the lower region for most participants, which may have had a greater distracting effect on participants in the ASD group with tactile sensitivities.

Furthermore, more and better-structured data of this form may still leave our understanding incomplete for fundamental ethological reasons. There remain many uncertainties about the motivational factors, neurocognitive processes, and temporal requirements of combined facial engagement and emotion recognition. These uncertainties make it a challenge to design an experiment with “just right” parameters, even now, when technology seems increasingly up to the task. For the work described in this study, to measure both emotion recognition and attention to faces simultaneously, we used video-presented static photos of children and had the children look at these photos for 6 seconds to be sure they had the maximum opportunity to perform at their best. Although the photos in the CAFE dataset are naturalistic and representative of diverse children, this experimental condition is quite divergent from real-life social interactions with moving children and fleeting displays of facial emotion. Furthermore, there are obvious differences between looking at the face of a child on a video screen and naturalistic facial engagement between two children. Given the differences between these experimental conditions and “real life,” the field still does not have enough information to know how divergent our experimental data (or another with different parameters attempted) might be from the ecologically valid situations.

### Future Outlook

To build a robust dataset toward at-home continuous phenotyping, the best course of action will be to capture the data at home as well. Key ingredients for building a robust dataset toward at-home continuous phenotyping are as follows: (1) large-scale in-home data acquisition, (2) augmented reality platforms integrating robust eye tracking, (3) game design for at-home analysis platforms, and (4) the use of clearly-defined and measurable experimental parameters [24]. We hope that in future studies, analyses on gaze and expression response in ASD can be made on data gathered in the home, in less-controlled settings, performed by families without the express need for specialist input. However, given that follow-up studies on the Superpower Glass system show heterogeneity in less-restricted data collection, this will likely be a challenge for effective human-computer interaction design: producing appropriate user interfaces and games that homogenize use in ways so as to lower the natural variability of measurement [28]. We expect data acquisition to be bolstered significantly by robust gaze-tracking hardware that can be developed at a low cost . Much of the low-cost gaze-tracking system envisioned for this study showed limitations once put on the children and had to be met with relatively expensive manual data recovery efforts. Further generations of the Superpower Glass system have not used the eye tracker in at-home studies because of its bulkiness and calibration issues [34,53]. As newer augmented reality devices entering the market begin to implement native gaze tracking, it is likely that these issues can be overcome.

## Appendix

Multimedia Appendix 1 Supplementary Gaze Tracking Implementation Details.

## Abbreviations

ABIQ: Abbreviated Battery Intelligence Quotient
ASD: autism spectrum disorder
CAFE: Child Affective Facial Expression
IRB: institutional review board
NC: neurotypical control
SCQ: Social Communication Questionnaire
SRS: Social Responsiveness Scale
