# Supplementary material for: Toward Continuous Social Phenotyping: Analyzing Gaze Patterns in an Emotion Recognition Task for Children With Autism Through Wearable Smart Glasses
Source: J Med Internet Res. 2020 Apr 22;22(4):e13810. doi: 10.2196/13810 (PMC7203617; doi:10.2196/13810)
Supplement: Multimedia Appendix 1 [file jmir_v22i4e13810_app1.docx]

**Towards Continuous Social Phenotyping: Analyzing Gaze Patterns in an Emotion Recognition**

# Multimedia Appendix 1: Supplementary Gaze Tracking Implementation Details

## Implementation

Pupil and calibration dot tracking was implemented in OpenCV in C++. We employed semi-automated approaches that combined several deterministic methods with manual validation to ensure accuracy in tracking both the pupil center and the calibration dot. The calibration procedure was designed to have considerable redundancy in range of motion. Hyperparameters of the various algorithms were manually tuned for videos with a high failure rate.

## Calibration Screen Dot Tracking

We employed standard temporal blob detection and various image processing techniques for smoothing. Upon successful identification of the blob, its motion was tracked in a local search window.

## Pupil Tracking

In a first pass, we utilized MSER and a gradient-based method to track pupil images. For videos that could not be successfully tracked in this way, we ran a semi-supervised optical flow algorithm. In a custom-built interface a labeler, given an eye image, annotated the location of the pupil (top-, bottom-, left-, and rightmost points). In subsequent frames, optical flow was used to match any frame with annotated points to the annotated frame. If the algorithm failed to find a match (for any of the annotations) for a given frame, the annotator was prompted to annotate that frame.

Pseudocode:

**for each frame:**

- **if we have no annotated frames, or we have failed to track
 for a sufficient number of frames:**

- query user to annotate current frame

- append resulting annotation (4 points) to end of cache

- add centroid of points as pupil position for that frame

- **try to track:**

- for each annotation in cache:

- try optical flow matching annotation to current frame

- if successful:

- add centroid of points as pupil

- position for that frame

- break

## World-Eye Calibration

Given calibration data in the form of pupil location $\{{p^{⃗}}^{(1)},{p^{⃗}}^{(2)},\ldots\}$and world point location $\{{w^{⃗}}^{(1)},{w^{⃗}}^{(2)},\ldots\}$, all $\in R^{2}$, we compute the feature vector

$f(p^{⃗})=(1,p_{x},p_{y},p_{x}^{2},p_{y}^{2},p_{x}p_{y},p_{x}^{2}p_{y}^{2})^{T}$

and fit a corresponding weight vector $\theta^{*}\in R^{7}$by solving the optimization problem

$\theta^{*}=arg\min_{\theta}\sum_{p^{⃗}corresponding tow^{⃗}}L_{\delta}(\theta^{T}f(p^{⃗})-w^{⃗})$,

where $L_{\delta}$ is the Huber loss function, which is robust to outliers. We performed this procedure iteratively (twice) and remove calibration outliers.

## Multiple Calibrations

As multiple calibrations were provided for each participant, a single labeler manually annotated which calibration appeared to be suited best to each block of frames to correct for unforeseen shifts of the eye tracker. The labeler validated whether such a shift occurred by paying attention to the frames displaying a dot in the center of the screen in regular intervals.

## Gaze Cluster Annotation

Three separate annotators labeled the eye positions of all participants throughout the computer task as either right (R), left (L), face (F), or nowhere (N) in particular on the displayed images (Figure A1).


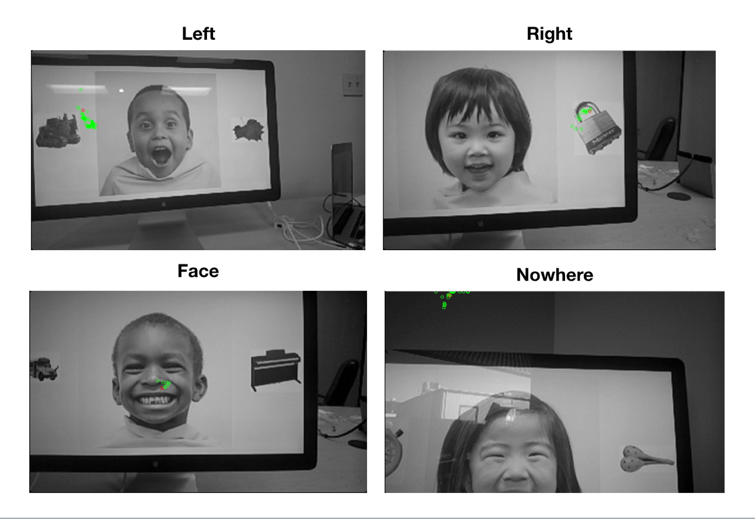


**Figure A1**. Examples of left, right, face, and nowhere clusters.

To label over 30,000 frames of rapid eye movement in bulk, we clustered points of continuous frames where the distance between age points was below a horizontal and vertical threshold (Figure A2).


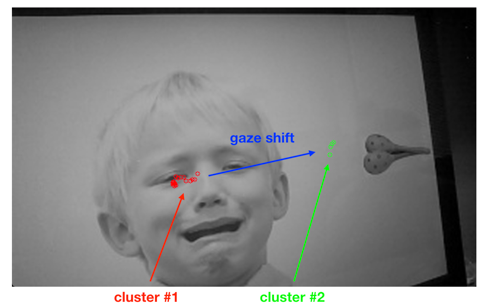


**Figure A2**. Visualization of radial and temporal clustering strategy.

Additionally, a limit was added to avoid too large lusters and a split function was created to split larger clusters into smaller gaze ones. Finally, annotators could manually split clusters that appeared too large.

Inter-rater reliability was assessed through confusion matrices and Cohen’s kappa coefficients. The confusion matrix is defined to be the square matrix with rows and columns corresponding to the possible gaze labels (L, R, F, N), with the entry in row r and column c to be the number of frames for which one annotators inputted a label corresponding to r but the other annotator inputted a label corresponding to c for the same frame. The confusion matrices were normalized such that each row sums to 1. Additionally, Cohen’s kappa coefficients, which account for agreement beyond occurrences of random chance, were calculated for each pair of annotators (Figure A3).


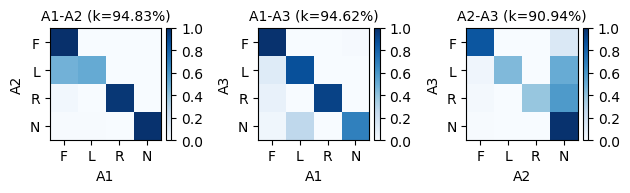


**Figure A3**. Normalized confusion matrices of gaze labels between three annotators (A1, A2, A3) with corresponding Cohen’s kappa coefficients.

## Outlier Removal

Several actions were taken to reduce the amount of potential uncertainty introduced into the gaze data with manual annotations. First, only the frames with a gaze label that both annotators agreed upon were used for further analysis. Second, only the frames with the most fundamental tags were used for analysis (F, L, R). The frames labeled as “nowhere” were associated with larger errors as seen in Figure A3. Two of the participants received irregular sequences and number of faces during the test administration, so they were removed from the original 33 participants.

To account for insufficient valid data for various participants, a two-step outlier analysis was performed. Outlier removal was performed on a per-trial basis, therefore, one participant’s data could be included in trial 1 and 2 analyses, but removed in trial 3 analysis. As a primary metric, the total number of frames with mutually agreed-upon gaze labels was considered. Only participants with a total frame number that lies below $Q1-1.5*IQR$, were deemed outliers. The next layer of removal involved the ratio of valid frames (L, R, F) to total frames to ensure that the captured data was not too diluted by invalid data. The number of valid frames is defined as the total number of frames with mutual agreement that do not include the nowhere tag. Ideally, this ratio would be closer to 1, indicating a large portion of valid frames amongst the total frames. The same procedure was used to remove those participants with ratios below $Q1-1.5*IQR$ (Figure A4).


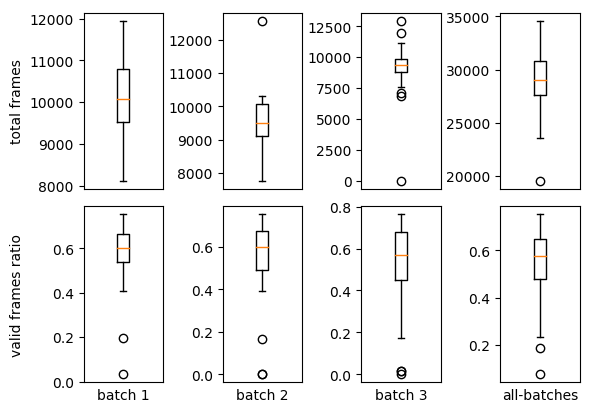


**Figure A4**. Distribution of total frames and valid frame ratios across combinations of trials considered in outlier removal.
